# Mechanisms and Therapeutic Prospects of Diabetic Cardiomyopathy Through the Inflammatory Response

**DOI:** 10.3389/fphys.2021.694864

**Published:** 2021-06-21

**Authors:** Namrita Kaur, Yingshu Guan, Rida Raja, Andrea Ruiz-Velasco, Wei Liu

**Affiliations:** Division of Cardiovascular Sciences, School of Medical Sciences, Faculty of Biology, Medicine, and Health, The University of Manchester, Manchester, United Kingdom

**Keywords:** diabetes mellitus, inflammation, diabetic cardiomyopathy, treatment, heart failure

## Abstract

The incidence of heart failure (HF) continues to increase rapidly in patients with diabetes. It is marked by myocardial remodeling, including fibrosis, hypertrophy, and cell death, leading to diastolic dysfunction with or without systolic dysfunction. Diabetic cardiomyopathy (DCM) is a distinct myocardial disease in the absence of coronary artery disease. DCM is partially induced by chronic systemic inflammation, underpinned by a hostile environment due to hyperglycemia, hyperlipidemia, hyperinsulinemia, and insulin resistance. The detrimental role of leukocytes, cytokines, and chemokines is evident in the diabetic heart, yet the precise role of inflammation as a cause or consequence of DCM remains incompletely understood. Here, we provide a concise review of the inflammatory signaling mechanisms contributing to the clinical complications of diabetes-associated HF. Overall, the impact of inflammation on the onset and development of DCM suggests the potential benefits of targeting inflammatory cascades to prevent DCM. This review is tailored to outline the known effects of the current anti-diabetic drugs, anti-inflammatory therapies, and natural compounds on inflammation, which mitigate HF progression in diabetic populations.

## Introduction

Diabetes mellitus (DM) is present in 40% of heart failure (HF) patients and is concomitant with increased hospitalizations and risk of mortality (Tromp et al., 2020). Inflammation is widely recognized to play a crucial role in the pathogenesis of both HF with reduced and preserved ejection fraction. Systemic inflammation is frequently associated with abnormal cardiac structure and function in clinical studies (Suzuki et al., 2008; Sanders-van Wijk et al., 2020): nevertheless, conflicting outcomes have also been documented. In the Canakinumab Anti-Inflammatory Thrombosis Outcomes Study (CANTOS) (Ridker et al., 2012), anti-interleukin (IL)-1β treatment demonstrated a reduction in inflammatory markers in type 2 DM (T2DM); however, over a longer duration, it failed to reduce the risk of cardiovascular events (Tan et al., 2020). Therefore, an in-depth understanding of inflammatory molecular mechanisms is needed to outline potential treatment strategies for managing inflammation in diabetes.

## Immune Signaling in the Heart

Myocardial injury instigated from myocarditis, myocardial infarction (MI), or metabolic stress triggers the innate and adaptive immune response in the heart. The innate response is a non-specific defense against cardiac injury, whereas the adaptive response is perpetuated by B and T cells designed to restore function (Mann, 2015). A pathological insult prompts the generation of pathogen-associated molecular patterns (PAMPs) or danger-associated molecular patterns (DAMPs) from cardiomyocytes, endothelial cells, fibroblasts, and leukocytes, depending on the stimuli (Castillo et al., 2020). Consequently, upon ligand binding, activation of DAMP/PAMP receptors and NLR family pyrin domain containing 3 (NLRP3) inflammasome pathways promote the production of pro-inflammatory cytokines, including tumor necrosis factor alpha (TNFα), IL-1β, IL-6, and IL-18, contributing to cardiac injury (Fairweather, 2007). The released inflammatory cytokines result in cardiac infiltration of leukocytes stimulating a restorative response in the heart. In various diseases, including diabetes, due to the lack of a resolution phase of the inflammatory state, myocardial inflammation contributes to pathological hypertrophic growth and leukocyte-mediated death of cardiomyocytes (Adamo et al., 2020). Besides, inflammatory cytokines also activate cardiac fibroblasts, inducing excessive interstitial fibrosis formation, leading to cardiac dysfunction (Franssen et al., 2016).

## Myocardial Inflammation in Diabetic Cardiomyopathy

Hyperinsulinemia, insulin resistance, hyperglycemia, and hyperlipidemia induce diabetic cardiomyopathy (DCM) consequently resulting in HF (Nunes et al., 2012). DCM is characterized by cardiomyocyte death, hypertrophy, and fibrosis, and these aberrant events are a consequence of pro-inflammatory cascades occurring in different cardiac cell types (Tan et al., 2020). Diabetes-induced alterations in endothelial (Knapp et al., 2019) and cardiac muscle cells (Filardi et al., 2019) are reported to be one of the major causative elements in the onset and progression of DCM. Specifically, this mini review focuses on the inflammatory mechanisms implicated in cardiomyocytes in diabetes.

A positive feedback loop emanates from the stress-induced release of pro-inflammatory molecules such as TNFα, IL-1β, and IL-6, accentuating leukocyte activation. This further activates the nuclear factor kappa-light-chain-enhancer of activated B cells (NF-κB), a transcription factor with antioxidant function in the physiological state (Maier et al., 2012). Under pathological conditions, such as DCM, over-activation of NF-κB results in more prominent leukocyte recruitment to the heart (Bajpai and Tilley, 2018). Abundant leukocyte infiltration was exemplified in immunohistochemical staining of right atrial tissue from T2DM patients, showing increased CD68^+^ macrophages compared to non-T2DM patients (Pierzynová et al., 2019). Moreover, diabetic stress can also induce hematopoiesis, resulting in increased circulating leukocytes (Nagareddy et al., 2013), fueling low-grade inflammation in the myocardium.

### The Different Roles of Leukocytes in Myocardial Inflammation

Leukocyte activation and recruitment are responsible for diabetic cardiac injury. Neutrophils are first-responders and secrete various inflammatory mediators, such as cytokines, microparticles, and neutrophil extracellular traps (NETs), which induce sustained inflammation. Of note, an increased neutrophil/lymphocyte ratio was recently identified as an indicator associated with subclinical DCM ocurrence (Huang et al., 2020). Furthermore, elevated NET release following protein arginine deiminase upregulation in neutrophils (Wong et al., 2015) aggravates cardiac injury due to neutrophil-mediated cell death (NETosis) in diabetes (Fadini et al., 2016).

Macrophages engulf apoptotic/necrotic cardiomyocytes and debris tomanage inflammation. However, this is impaired in diabetes due toreduced miR-126 expression and blunted MERTK function(Suresh Babu et al., 2016; Bajpai and Tilley, 2018). Moreover, macrophages areclassified as pro-inflammatory (M1) and anti-inflammatory (M2), characterized by distinct sources of activation. The phenotypicbalance between these subsets is necessary for the homeostasis ofinflammatory responses. M1 macrophages arise from IFN-γ and secrete IL-6, TNFα, IL-1β, IL-12, and IL-23, whereas M2 macrophages are polarized by IL-4, IL-10, or IL-13 and express IL-10 and transforming growth factor beta (TGFβ) (Van Linthout and Tschope, 2017). M1 macrophages are more predominant in diabetes, instigating insulin resistance by secreting resistin and prompting DCM progression (Lehrke et al., 2004); however, the M2 phenotype ameliorates cardiac dysfunction in DM (Jadhav et al., 2013). For instance, elevated M2 macrophage differentiation mitigated heart dysfunction following fibroblast growth factor (FGF)-9 (Singla et al., 2015) and bone morphogenetic protein 7 (BMP-7) (Urbina and Singla, 2014) treatment in diabetic rodents.

In the adaptive response, T helper (Th)-1 or Th-17 cells secrete pro-inflammatory cytokines, whereas T regulatory (Treg) cells secrete anti-inflammatory cytokines. In T2DM patients, skewed Th/Treg balance and elevated T cell homing contribute to cardiovascular complications by increasing cardiac hypertrophy and fibrosis (Zeng et al., 2012; Nevers et al., 2015; Bajpai and Tilley, 2018). Moreover, increased cytokines from systemic Th17 are associated with diastolic abnormality in diabetic children (Hoffman et al., 2013). Evidently, myocardial T-cell infiltration induces fibrosis in type 1 DM (T1DM) mice via increased TGFβ expression, which is diminished by T-cell depletion (Abdullah et al., 2016). Lastly, B-cells maintain the bridge between innate and adaptive immunity via their antigen-specific response, and their presence contributes toward sustained inflammation in DCM. For instance, overexpression of allograft inflammatory factor (AIF-1), an anti-inflammatory cytokine, prevents streptozotocin (STZ)-induced cardiac dysfunction; on the contrary, AIF-1 downregulation is associated with elevated B-cell homing in the myocardium (Sarkar et al., 2018). The role of inflammatory cytokines, chemokines, and receptors in DCM is outlined in Table 1.

**TABLE 1:** The role of inflammatory cytokines, chemokines, and receptors in DCM.

	**Name**	**Role**	**Pre-clinical findings**	**Clinical findings**	**References**
**Cytokines**	TNFα	Pro-inflammatory	STZ-induced diabetic rats with anti-TNFα antibody treatment: improved LV function, ↓IL-1β expression, and ↓cardiac collagen content.	↑Plasma TNFα level is associated with LV diastolic dysfunction in patients with diabetes	Westermann et al., 2007a, b; Dinh et al., 2009
	IL-18	Pro-inflammatory	IL-18 KO mice fed with western diet: preserved cardiac function and ↓myocardial interstitial fibrosis	↑IL-18 level is an independent predictor of CV events in patients with metabolic syndrome	Troseid et al., 2009; Carbone et al., 2017
	IL-6	Pro-inflammatory	IL-6 KO mice with STZ-induced diabetes: improved cardiac function and ↓interstitial fibrosis; ↓TGFβ and ↑miR-29 following high glucose	↑Plasma IL-6 level is associated with LV diastolic dysfunction in patients with diabetes	Dinh et al., 2009; Zhang et al., 2016
	TGFβ	Fibrogenic mediator, pro-inflammatory	*Db/db* Smad3+/−mice: attenuated cardiac diastolic dysfunction, ↓hypertrophy, and fibrosis	↑Serum TGFβ level is associated with diastolic dysfunction in hypertensive patients with metabolic syndrome	Sciarretta et al., 2007; Biernacka et al., 2015
	IL-1β	Pro-inflammatory	STZ-induced diabetic mice: ↑cardiac IL-1β expression, ↑cardiac collagen content, and LV dysfunction; also associated with cardiac arrhythmias	Canakinumab (a human monoclonal antibody that neutralizes IL-1β) reduces CRP level and cardiovascular events in patients with or without T2DM	Westermann et al., 2007a, b; Ridker et al., 2012; Monnerat et al., 2016
	HMGB1	Pro-inflammatory	Hyperglycemia induces ↑HMGB1 expression and NF-κB activity in the heart. STZ-induced diabetic mice with HMGB1 silencing: ameliorated LV dysfunction and remodeling	↑Serum HMGB1 in patients with diabetes with HF; HMGB1 levels inversely related to LV ejection fraction in HF patients with or without diabetes	Volz et al., 2010; Wang et al., 2011; Wang W. et al., 2014
**Chemokines**	MCP-1	Stimulates monocytes and macrophages	MCP-1 induces glucose-mediated cell death in isolated cardiomyocytes via oxidative and endoplasmic-reticulum stress	↑Plasma MCP-1 level in T2DM patients associated with CV-associated mortality	Piemonti et al., 2009; Younce et al., 2010
	MMP-2	ECM degradation	STZ-induced diabetic mice: ↓MMP-2 and ↑Smad7 expression contribute to cardiac fibrosis	↑Serum MMP-2 level in patients with and without diabetes; not an independent risk factor	Van Linthout et al., 2008; Kobusiak-Prokopowicz et al., 2018
**Receptors**	CCR2	Macrophage recruitment	CCR2 KO in STZ-induced diabetic and CCR2 inhibition in *db/db* mice: improved cardiac dysfunction, ↓oxidative stress, and M1 macrophage infiltration along with reversing hyperglycemia	↑CCR2 expression of circulating monocytes associated with ↑arterial wall inflammation in patients with high risk of CV event, including patients with diabetes	Verweij et al., 2018; Tan et al., 2019
	RAGE	Pro-inflammatory	RAGE KO mice fed a high-fat diet: ↓Cardiac hypertrophy, inflammation, and collagen accumulation due to ↓oxidative stress	↑Serum cRAGE and HMGB1 levels in diabetic HF patients vs. non-diabetic HF patients; associated with development and severity of HF	Tikellis et al., 2008; Wang et al., 2011
	TLR (4,2)	Pro-inflammatory	STZ-induced diabetic mice with TLR4 silencing: ↓fibrosis and expression of TGFβ and adhesion molecules, preserves cardiac contractility Stimulation of TLR-2 in HL-1 cardiomyocytes ↑NF-κB activation, thereby decreasing contractility	TLR4 mutation confers protection against T2DM, but not against ischemic heart diseases in diabetic and non-diabetic patients	Boyd et al., 2006; Manolakis et al., 2011; Zhang et al., 2020

*CCR2, C-C motif chemokine receptor 2; cRAGE, cleaved RAGE; CRP, C-reactive protein; CV, cardiovascular; ECM, extracellular matrix; HF, heart failure; HMGB1, high-mobility group box protein 1; IL, interleukin; KO, knockout; LV, left ventricle; MCP-1, monocyte chemoattractant protein-1; MMP-2, matrix metalloproteinase-2; NF-κB, nuclear factor kappa-light-chain-enhancer of activated B cells; RAGE, receptor for advanced glycation end products; STZ, streptozotocin; TGFβ, transforming growth factor beta; TLR, Toll-like receptor; TNFα, tumor necrosis factor alpha; T1DM, type 1 diabetes mellitus; T2DM, type 2 diabetes mellitus; ↑, increased; ↓, decreased.*

### NF-κB-Associated Signaling Pathways

Overactivation of molecular pathways, such as NF-κB, signal transducer activating protein-1 (AP-1), c-Jun NH_2_-terminal kinase (JNK), and p38 mitogen-activated protein kinase (MAPK), favors the induction of a pro-inflammatory intramyocardial milieu in DM. NF-κB pathway is the central converging point of inflammatory triggers stemming from several pathological stresses in diabetes, such as prolonged endoplasmic reticulum stress, hyperlipidemia, hyperglycemia, renin–angiotensin–aldosterone system (RAAS) activation (Knapp et al., 2019), oxidative stress (Maier et al., 2012), and advanced glycation end products (AGEs) (Palomer et al., 2013). NF-κB activation contributes to myocardial fibrosis, hypertrophy, apoptosis, and ventricular dysfunction (Frati et al., 2017). On the other hand, NF-κB inhibition by IκB-α overexpression showed reduced RAAS activation and preserved calcium handling in STZ-induced diabetic heart (Thomas et al., 2014). ROS production in response to hyperlipidemia, hyperglycemia, and mitochondrial dysfunction also triggers NF-κB signaling in the diabetic heart via degradation of IκB-α (Baker et al., 2011) and downregulation of nuclear factor erythroid 2-related factor 2 (Nrf-2) following Erk1/2 activation (Tan et al., 2011). Moreover, elevated DAMP release from isolated cardiomyocytes, macrophages, fibroblasts, and endothelial cells under diabetic conditions (Frati et al., 2017) results in NF-κB activation accompanied by increase in cytokines (Wang W. et al., 2014). In T1DM, NF-κB activity is increased following hyperglycemia, oxidative stress-induced JNK phosphorylation (Pan et al., 2014), and reduction in insulin-induced GSK-3β phosphorylation (Wang et al., 2009; Ge et al., 2019), thereby augmenting lipid accumulation, inflammation, and fibrosis in the heart. Furthermore, high glucose-mediated post-translational modifications in the p65 subunit of NF-κB alter its activity. For example, p65 O-GlcNAcylation enhances NF-κB activation by decreasing its binding to IκB-α (Yang et al., 2008); on the contrary, sirtuin 1-mediated p65 deacetylation at a lysine site (Lys310) suppresses NF-κB transcriptional activity (Planavila et al., 2011), thereby modulating cardiac inflammation.

### Roles of the Receptor for AGE- and TLR-Mediated Cardiac Inflammation

Advanced glycation end products are generated from non-enzymatic glycation and oxidation of proteins or lipids in response to metabolic stress (Ahmed, 2005). The receptor for AGEs (RAGEs) binds multiple ligands, including AGEs and DAMPs (Ramasamy et al., 2011), contributing to the generation of pro-inflammatory cytokines and oxidative stress (Koyama et al., 2008). Elevated AGE formation is correlated with collagen accumulation, myocardial fibrosis, impaired calcium homeostasis, and mitochondrial dysfunction in the diabetic heart (Bidasee et al., 2003). Moreover, Toll-like receptors (TLRs) are essential to activate innate immunity by responding to PAMPs or DAMPs and participate in adaptive immunity by regulating the activation of circulating lymphocytes (Mann, 2011). Increased free fatty acid (FFA) levels in diabetes promote inflammation via AGE production and activation of TLR4 (Kim et al., 2007) and protein kinase C (PKC) (Itani et al., 2002), resulting in increased NF-κB activity. Furthermore, TLR4-mediated inflammatory signaling is apparent in several animal models of T1DM (Tan et al., 2020), such that genetic ablation of TLR4 reduced cardiac inflammation and improved cardiac function (Dong et al., 2012). The hetero-dimerization of RAGE and TLR stimulated pro-IL-1β and pro-IL-18, whereas impediment of RAGE reduced the cardiac inflammatory response in *db/db* mice and improved diastolic function (Nielsen et al., 2009). Interestingly, the interaction of AGEs with RAGE also triggers NF-κB activation, further transcriptionally regulating RAGE expression in a positive feedback loop (Gao et al., 2008) and aggravating cardiac inflammation. Hyperglycemia-induced AGEs can also directly bind to myeloid differentiation 2 (MD2)–TLR4 receptor complex, initiating inflammatory pathways and consequent myocardial injury, contributing to cardiac dysfunction in type 1 and type 2 diabetic mice (Wang et al., 2020).

### Involvement of NLRP3 Inflammasome in Cardiac Inflammation

**FIGURE 1:**
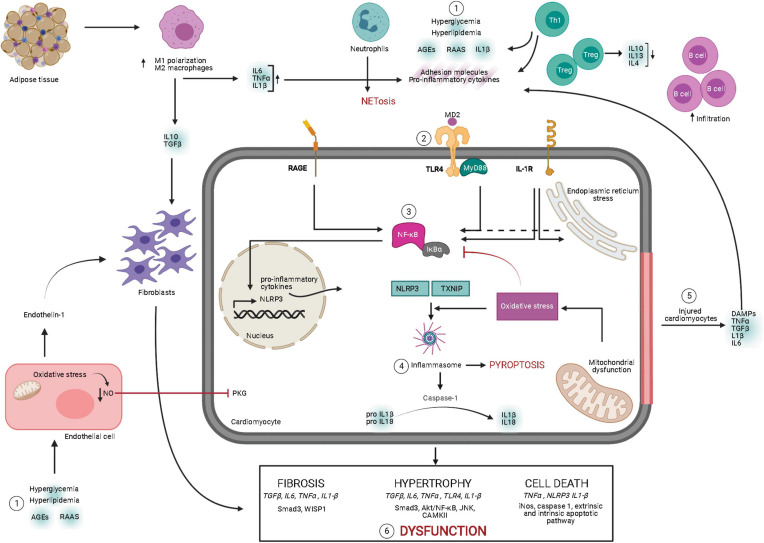
Overview of signaling mechanisms underlying myocardial inflammation in diabetes mellitus. Diabetic milieu comprises of elevated leukocyte homing in the myocardium. Pathological stresses such as hyperglycemia, hyperlipidemia, elevated RAAS, and AGEs induce secretion of pro-inflammatory molecules, adhesion molecules, and DAMPs from the leukocytes. Moreover, these instigators also induce ROS-mediated endothelial dysfunction contributing to cardiac remodeling. Secreted pro-inflammatory cytokines bind to the receptors, such as TLR-4–MyD88 complex, RAGE, and IL-1R, and initiate their intracellular signaling pathways. These pathways activate NF-κB, resulting in transcriptional upregulation of inflammatory cytokines and NLRP3 inflammasome. Following NF-κB activation and oxidative stress, inflammasome assembly leads to maturation of IL-1β and IL-18, along with induction of pyroptosis. Meanwhile, stressed or injured cardiomyocytes release pro-inflammatory cytokines and DAMPs, contributing to aggravated inflammatory cascades. Chronic inflammatory cytokine-induced intracellular response leads to pathological cardiac remodeling and cardiac dysfunction. AGE, advanced end glycation products; DAMP, danger-associated molecular pattern; IL, interleukin; IL-1R, interleukin 1 beta receptor; MD2, myeloid differentiation 2; MyD88, myeloid differentiation primary response 88; NLRP3, NLR family pyrin domain containing 3; NETosis, neutrophil-mediated cell death; NF-κB, nuclear factor kappa-light-chain-enhancer of activated B cells; NO, nitric oxide; PKG, protein kinase G; RAAS, renin–angiotensin–aldosterone system; RAGE, receptor for AGEs; TGFβ, transforming growth factor beta; TLR-4, Toll-like receptor 4; TNFα, tumor necrosis factor alpha; TXNIP, thioredoxin-interacting/inhibiting protein (created with Biorender.com).

Inflammasome stimulation is a two-step process requiring priming by inflammatory stimuli. The first step is NF-κB transcriptional upregulation of NLRP3 and pro-IL-1β. The second step involves DAMP-mediated inflammasome assembly, causing oxidative stress and inflammation-induced programmed cell death, also known as pyroptosis (Luo et al., 2017). In diabetes, mitochondrial damage has been detected as an important contributor to inflammasome assembly through the release of mitochondrial DNA and ROS. Moreover, excessive cytokines, in turn, exacerbate mitochondrial dysfunction in a positive feedback loop (Luo et al., 2014; Schilling, 2015). Interestingly, inflammasome expression is markedly increased in rodent diabetic hearts via oxidative stress-dependent thioredoxin-interacting/inhibiting protein (TXNIP) activation, showing elevated caspase-1 and IL-1β activation (Stienstra et al., 2011). This pro-inflammatory mechanism is absent in high-fat diet-fed mice with NLRP3 deficiency (Luo et al., 2014). NLRP3 inflammasome cleaves caspase-1 from pro-caspase 1, which is involved in the maturation of inflammatory cytokines, such as IL-1β, and pyroptosis-triggered fibrosis in DCM (Peiró et al., 2017). IL-1β triggers multiple signaling pathways through its interaction with the IL-1β receptor on cardiomyocytes in diabetic conditions (Dinarello, 2009). For instance, in T1DM, IL-1β promoted C/EBP homologous protein (CHOP)-dependent cell death and cardiac dysfunction, which is attenuated by the administration of recombinant IL-1 receptor antagonist (Liu et al., 2015). Similarly, pharmacological inhibition of caspase-1 attenuated inflammation and cardiac dysfunction in STZ-injected rats (Westermann et al., 2007a). The schematic representation of inflammatory signaling in DCM is shown in Figure 1.

## Therapeutic Strategies for Prevention of DCM by Targeting Inflammation

Given the intimate association between inflammation and DCM, therapeutic interventions targeting myocardial inflammation are essential to mitigate the onset and progression of HF in patients with DM. First, the inflammatory cascade instigators, such as hyperglycemia, hyperlipidemia, oxidative stress, and insulin resistance, continuously aggravate inflammation following the onset of DCM. Therefore, managing these instigators could aid to regulate inflammation and prevent HF development. Second, the inflammatory signaling processes can be modulated directly to prevent cardiac dysfunction in diabetes. For instance, suppression of inflammation is achieved by inhibition of pro-inflammatory cytokines, chemokines, and DAMPs; macrophage polarization toward M2 phenotype; moderation of inflammasome activity; and restraint of leukocyte recruitment.

### Anti-diabetic Drugs

#### Insulin Sensitizers

Current anti-diabetic therapies improve glycemic control and insulin sensitivity in patients with diabetes and thereby indirectly manage systemic and myocardial inflammation (Reis et al., 2012). Abrogation of cardiac insulin resistance is shown to mitigate inflammatory cardiac dysfunction by decreased production of pro-inflammatory adhesion molecules, C-reactive protein (CRP), and IL-6 (Dandona et al., 2009; Al-Huseini et al., 2019). Metformin, the first-line anti-diabetic drug in clinics, promotes glucose homeostasis and improves impaired heart function in patients with diabetes by blocking pro-inflammatory markers such as CCL11 (Dludla et al., 2020). Notably, metformin also exhibits its anti-inflammatory effects by inhibiting NF-κB (Hattori et al., 2006), reducing CRP production from vessel walls (Li et al., 2009), and blocking monocyte differentiation into macrophages (Vasamsetti et al., 2015), irrespective of diabetic status. However, the mechanisms of metformin’s anti-inflammatory action in DCM require further attention.

Peroxisome proliferator-activated receptor-γ (PPARγ) is a member of the PPAR nuclear hormone receptor superfamily, and its activation promotes pleiotropic biological effects such as reduced serum glucose and regulated cardiac metabolism in DM (Liu et al., 2016). The use of a PPARγ agonist, rosiglitazone, has shown anti-inflammatory effects in T2DM patients resulting in improved diastolic function (von Bibra et al., 2008). In contrast, other insulin-sensitizing agents and thiazolidinediones present a neutral or deleterious effect on cardiac structure and function in T2DM patients (Naka et al., 2010), making their anti-inflammatory role unsubstantiated in DCM.

#### Sodium Glucose Co-transporter 2 Inhibitors

Sodium–glucose co-transporter 2 (SGLT2) inhibitors (SGLT2is) promote glycosuria by inhibiting SGLT2 in nephrons and thereby improve glycemic control (Kenny and Abel, 2019). More recently, SGLT2is have gained attention due to their ability to reduce HF progression in patients irrelevant of their diabetic status (Natali et al., 2017; Zannad et al., 2020). In diabetic patients, SGLT2is likely potentiate their cardioprotective effects through multiple actions, including amelioration of inflammation (Scheen, 2020). SGLT2is are able to reduce endothelial inflammation in T1DM mice (Zhou et al., 2018), decrease cardiac macrophage infiltration in pre-diabetic rats (Kusaka et al., 2016), downregulate cardiac cytokine expression (Radlinger et al., 2020), and attenuate inflammasome activation (Ye et al., 2017), all of which result in repressed secretion of pro-inflammatory cytokines and improved cardiac function in T2DM genetic models (Aragon-Herrera et al., 2019). Furthermore, STZ-injected rats displayed decreased cardiac expression of NLRP3, caspase-1, and IL-1β following SGLT2i (empagliflozin) treatment (Trang et al., 2021). SGLT2is are suggested to alleviate cardiac inflammation independent of their anti-hyperglycemic effect observed via reducing TLR4 and TNFα in angiotensin-II-induced cardiomyopathy in *db/db* mice (Arow et al., 2020). Taken together, these studies suggest that SGLT2is exert anti-inflammatory effects that positively influence cardiac function in rodent models of DM; however, the mechanistic link remains poorly defined. Of note, the majority of data have been obtained from T1DM pre-clinical models; therefore, the role of anti-diabetic drugs on cardiac inflammation in T2DM needs further exploration.

#### Dipeptidyl Peptidase 4 Inhibition and Glucagon-Like Peptide-1 Agonists

Dipeptidyl peptidase 4 (DPP-4) inhibitors increase incretin hormone, glucagon-like peptide 1 (GLP-1) levels exerting beneficial actions on glucose homeostasis and insulin sensitivity. Both DPP-4 inhibitors and GLP-1 agonists are used in clinics as anti-diabetic therapies; however, their role in protecting against cardiac dysfunction in diabetes remains uncertain. Linagliptin, a DPP-4 inhibitor, prevented cardiac dysfunction by attenuating inflammasome activation in *db/db* mice following MI. Moreover, *in vitro* experiments displayed a lower TLR4 in human cardiomyocytes and cardiofibroblasts following high-glucose stimulation and linagliptin treatment. Interestingly, this protective mechanism was absent following exenatide exposure, which is a GLP-1 analog, suggesting that DPP-4 inhibitors might have a direct anti-inflammatory effect regardless of GLP-1 levels (Birnbaum et al., 2019). Contrastingly, exendin-4, another GLP-1 analog, displayed cardioprotective effects via enhancing AMP-activated kinase (AMPK) phosphorylation following hyperglycemia in high-fat diet-fed mice (Wei et al., 2019). Also, DPP-4 inhibition reduces monocyte recruitment to the myocardium (Shah et al., 2011) and suppresses the activation of inflammatory proteases, thereafter preventing adverse cardiac remodeling (Kolpakov et al., 2019) in experimental models of DCM (Zhong et al., 2015). On the contrary, DPP-4 inhibitors also increase endogenous stromal cell-derived factor (SDF) (Packer, 2018), a chemokine emanating from adipose tissue, which promotes inflammation-induced fibrosis in the diabetic myocardium, albeit diminished by SDF receptor (CXCR4) antagonism (Chu et al., 2015). These conflicting results require further investigation to establish their anti-inflammatory potentials in DCM.

### Anti-inflammatory Therapies

Direct immune modulation can also be beneficial in the management of DM’s chronic inflammatory state. In T1DM mice, administration of FTY720 inhibited cardiac fibrosis by regulating T-cell infiltration (Abdullah et al., 2016). Nonetheless, targeting a single inflammatory mechanism might provoke a secondary compensatory inflammatory response. For instance, anti-TNFα therapy (Chung et al., 2003) or IL1β suppression (Torre-Amione et al., 2008) aggravated clinical outcomes in patients administered with the highest doses, owing to their inadvertent or unknown effects such as intrusion of homeostatic inflammation and agonist activity of the antagonists (Mann, 2015).

Immunosuppressants such as methotrexate improved myocardial inflammation via reduced expression of macrophages in T1DM rats (Cavalcante Maranhao et al., 2017). Methotrexate also reduced cardiovascular events in patients, though research is limited to patients with rheumatoid arthritis (Baghdadi, 2020). Lastly, adjunct anti-inflammatory therapies, such as statins and canakinumab, are suggested to reduce the burden of DCM, possibly by alleviating IL1β-dependent insulin resistance in metabolic disorders (Gao and Ye, 2012; Liberale et al., 2019). Overall, the current anti-inflammatory therapies seem promising yet require further exploration in the setting of DCM.

### Natural Compounds

The study of anti-inflammatory effects of natural compounds in DCM is on the rise in pre-clinical and clinical research. An adequate diet has demonstrated a significant implication for maintaining cardiac function in patients with diabetes (Kozlowska, 2017; Allen et al., 2019). For instance, quercetin, a flavonoid, lowers systolic blood pressure in type 2 diabetic women without a positive effect on inflammatory biomarkers contradictory to pre-clinical data (Rivera et al., 2008; Roslan et al., 2017), possibly due to small sample size recruitment (Zahedi et al., 2013). However, administration of curcumin, a turmeric root extract, lowers circulating pro-inflammatory markers in patients with diabetes-induced organ dysfunction (Gupta et al., 2013). Yet, its specific role in DCM patients remains to be elucidated.

Leukocyte infiltration is one of the early inflammatory events in DCM before the advent of clinical outcomes. There is compelling evidence that certain natural compounds such as isoliquiritigenin (ISL) and ginger extract can reduce macrophage infiltration via suppressed MAPK signaling (Gu et al., 2020) and reduced TGFβ expression (Abdi et al., 2021), respectively, in T1DM. However, only ISL preserved cardiac function; the ginger extract majorly prevented myocardial structural damage.

Certain natural compounds can ameliorate cardiac inflammation by directly modulating inflammatory responses. Probiotics, fungi, and medicinal plants exert an anti-inflammatory action via downregulation of TLR4 expression (Chiang et al., 2021) and reduction of IL-1β, TNFα, MCP-1, and TGFβ (Wang et al., 2018; Shaher et al., 2020), subsequently preserving cardiac structure and function in T1DM rodents. Moreover, hederagenin, a plant extract, improves cardiac function by diminishing secretion of pro-inflammatory cytokines and decreasing NF-κB transcriptional activity in *db/db* mice (Li Y. et al., 2019). Also, PPARγ agonism by crocin, a carotenoid compound found in saffron, reduces TNFα and IL-6 levels in diabetic rats following MI (Badavi et al., 2020). Similar anti-inflammatory effects were observed in clinical trials involving type 2 patients, which is likely due to the alleviation of insulin resistance and restoration of glycemic control; however, further research is warranted (Behrouz et al., 2020).

The anti-inflammatory effects of natural compounds are multifold, though varied among cell types. For instance, syringaresinol (SYR), a cereal extract, suppressed both Kelch-like ECH-associated protein 1 (KEAP1)/Nrf2 and TGFβ/Smad pathway in neonatal cardiomyocytes, resulting in reduced cardiac macrophage density and improved cardiac function (Li et al., 2020). Interestingly, SYR can also downregulate NF-κB activation via p38 stimulation in macrophages *in vitro*, thereby reducing inflammation indirectly (Bajpai et al., 2018). Some pleiotropic agents, such as curcumin (Zheng et al., 2018), sophocarpine (Zou et al., 2019), and luteolin (Li L. et al., 2019), ameliorate DCM by suppressing NF-κB signaling pathway and the subsequent secretion of pro-inflammatory molecules. In addition, curcumin analogs, C66 and J17, have both direct and indirect anti-inflammatory roles. C66 lowers serum and cardiac triglyceride levels and inhibits JNK signaling (Wang Y. et al., 2014). Moreover, J17 protects diabetic mice and H9c2 cardiomyoblasts against high glucose-induced inflammation by inhibition of p38 signaling pathway (Chen et al., 2017). Interestingly, both SYR-activated p38 in macrophages (Bajpai et al., 2018) and curcumin-inhibited p38 in cardiomyocytes modulate cardiac inflammation in diabetes. These opposing mechanistic effects encapsulate the complexity and challenge of targeting inflammatory mechanisms in DCM. Overall, these natural compounds effectuate anti-inflammatory properties in the heart, yet their mechanistic role in different cardiac cell types, long-term implications, and clinical relevance remain undetermined.

## Conclusion

Myocardial inflammation is a significant causative factor in diabetes-induced cardiac dysfunction. Positive feedback loops between the defective cardiomyocytes and harmful inflammatory responses lead to excessive pro-inflammatory cytokines and the recruitment of inflammatory cells in the myocardium, resulting in cardiac dysfunction. Clinical and pre-clinical studies demonstrate that mitigation of myocardial inflammation is closely linked to preserved cardiac function. Therefore, further efforts should be made to better understand the molecular mechanisms whereby cardiac inflammation contributes to DCM progression. Furthermore, a notable amount of research is still required to evaluate and develop therapeutic strategies targeting myocardial inflammation in diabetes.

## Author Contributions

NK and YG collected the references and generated the table. NK, YG, RR, and AR-V generated the figure and drafted and proofread the manuscript. NK and WL designed the manuscript. All authors contributed to the article and approved the submitted version.

## Conflict of Interest

The authors declare that the research was conducted in the absence of any commercial or financial relationships that could be construed as a potential conflict of interest.
